# The Spatio-Temporal Disparities of Areas Benefitting from the Wind Erosion Prevention Service

**DOI:** 10.3390/ijerph15071510

**Published:** 2018-07-17

**Authors:** Jie Xu, Yu Xiao, Gaodi Xie, Lin Zhen, Yangyang Wang, Yuan Jiang

**Affiliations:** 1 Institute of Geographic Sciences and Natural Resources Research, Chinese Academy of Sciences, Beijing 100101, China; xuj.16b@igsnrr.ac.cn (J.X.); xiegd@igsnrr.ac.cn (G.X.); zhenl@igsnrr.ac.cn (L.Z.); wangyy.15s@igsnrr.ac.cn (Y.W.); 2 College of Resources and Environment, University of the Chinese Academy of Sciences, Beijing 100049, China; 3 Faculty of Geographical Science, Beijing Normal University, Beijing 100049, China; jiangy@bnu.edu.cn

**Keywords:** ecosystem service flow, wind erosion prevention, service beneficiary areas, Yanchi County

## Abstract

Ecosystem services are closely linked to human welfare. The flow of ecosystem service can establish spatio-temporal relationships between ecosystem service provision areas (SPAs) and service beneficiary areas (SBAs). In this study, the Hybrid Single Particle Lagrangian Integrated Trajectory (HYSPLIT) model was used to simulate the spatial flow path of the wind erosion prevention (WEP) service in Yanchi County. The frequency at which the simulated trajectories passed through each grid cell was used as a proxy for spatio-temporal disparities in SBAs, and the distribution of benefitting land cover, population, and gross domestic product (GDP) reflected the effects of the WEP flow. The flow paths of the Yanchi County WEP in 2010 mainly extended to eastern and central China, North Korea, South Korea, Japan, Mongolia, and eastern Russia, and were more intensive and longer in spring and winter than in autumn and winter. The SBAs covered an area of 1153.2 × 10^4^ km^2^ in 2010, with dominant service beneficiary areas (DSBAs) comprising 185.1 × 10^4^ km^2^ and accounting for 16.1% of the total beneficiary area of the year. The areas through which the flow paths passed with a high frequency (≥10%) were mainly located in Shaanxi, Shanxi, Henan, western Shandong, Hebei, Beijing, and northern Hubei, and the spatial scale of these areas varied, demonstrating obvious seasonal changes, and was the largest in spring. The benefitting land cover was mainly cropland across all of the SBAs, with one billion benefitting people (accounting for 77.11% of the total population of China) associated with a gross domestic product (GDP) of 26.8 trillion RMB (Chinese currency; as of 2018-06-22, 6.497 RMB = US $1, accounting for 87.90% of the total GDP of China). Furthermore, the population and socio-economic development in the DSBAs (21 million people and 0.53 trillion RMB GDP) were no longer affected by wind erosion from Yanchi County. This study revealed the spatio-temporal disparities of the SBAs of WEP in Yanchi County from an ecosystem services flow perspective and provides a scientific and effective basis for policymakers to perform standard ecological compensation accounting and to formulate ecological protection policies.

## 1. Introduction

Human health is closely related to ecosystem services (ESs). ESs refer to the benefits that humans derive from nature, and increasingly emphasize the relationship between the environment and human welfare. Environmental risks cause 23% of all deaths globally and particularly affect children under 5 years old and adults aged 50–75 years old [1]. Most studies about the topic have focused on the human health threats posed by environmental risk distributions, with recent studies focusing on the positive contribution of ESs to health [2]. Strengthening the connection between human activities and ESs in nature can improve immune system function and mood, and reduce pressure and the incidence of non-communicable diseases (NCDs), such as respiratory disease [3].

Wind erosion prevention (WEP) is one of the most important ESs in wind erosion areas, and is a kind of protective service that includes the inhibition and fixation of vegetation on wind and sand [4], and can create conditions for the sustainable development of regional production and living. Wind erosion is a process in which the airflow that travels at a certain speed acts on soil or the parent material of soil, moving soil particles and thereby damaging the soil structure and causing a loss of soil material. Wind erosion is the first step of land desertification in arid, semi-arid, and some semi-humid regions. In addition, wind erosion is also the source of sand and dust (SD) storms in downwind regions, which adversely affect air quality in downwind cities, severely harm the health and quality of life of city residents, and increase the incidence of chronic respiratory illnesses [5]. WEP can prevent damage to human well-being and the social economy, including human health, traffic, communication, electricity, mining, construction, irrigation, and other facilities; in addition, WEP can reduce damage to the natural environment by, for example, reducing nutrient loss from soil. Therefore, determining the impact zone of wind erosion and the SBAs of WEP are of great importance to regional ecosystem protection, human health, and sustainable social and economic development.

The reduction of SD weather in WEP SBAs is the most direct manifestation of the effect of WEP flow. Therefore, the identification of WEP SBAs can be conducted on the basis of the wind erosion affected areas downwind. The flow paths of WEP extend from the sand source area (the service provision areas of WEP, i.e., SPAs) to the sand settling area (the service beneficiary areas of WEP, i.e., SBAs). The existing simulation models of SD movement are mostly air quality models, primarily the Weather Research and Forecasting model coupled with chemistry (WRF)-Chem [6], WRF-Dust, Global Environmental Multiscale (GEM) model and its developed GEM-AQ/EC [7], Community Atmosphere Model (CAM), Models-3/Community Multiscale Air Quality (CMAQ) [8], and the Hybrid Single Particle Lagrangian Integrated Trajectory (HYSPLIT) models [9]. The simulation accuracies of WRF-Chem, WRF-Dust, GEM-AQ/EC, CAM, and Models-3/CMAQ, which require meteorology, fluid mechanics, and other professional knowledge backgrounds, are much higher, and the accuracy requirements of the meteorological and pollution data sources of these models are high and of low operability, leading to high simulation time resource costs, which render them inapplicable in the interdisciplinary research field of ecosystem service flow and inefficient for WEP eco-compensation decision making. However, the HYSPLIT model, which can be obtained from the Air Resources Laboratory (ARL) at the National Oceanic and Atmospheric Administration (NOAA), is a modelling system with wide computational capabilities, ranging from simple trajectory to complex dispersion and deposition simulations, which has been widely used to study the regional transport and diffusion of many kinds of pollutants [10,11,12], including SD storms [13,14,15], particle matter (PM_2.5_) [16,17,18], tropospheric ozone, sulphur dioxide, benzene [19], volcanic ash and gases [20], forest fire pollutants, and mercury. Although the common resolution of the model is 1° × 1°, which can cause uncertainty in the study of small-scale meteorological systems such as land-sea breeze circulation, mountain-valley wind, and the urban heat island effect. However, HYSPLIT is generally good at analysing the long-range transport paths of pollutants. Because of its good feasibility, HYSPLIT is widely used in the study of the transregional diffusion of pollutants. Xiao et al. [21] analysed the flow paths, beneficiary areas, and effects of WEP in the Hunshandake by using the backward trajectory analysis of HYSPLIT. Rashki et al. [15] examined the seasonality of and areas affected by dust storms originating from the Sistan region, southeastern Iran, in the summer, by using HYSPLIT forward trajectories.

The case study region, Yanchi County, is in the eastern part of Ningxia. Most regions of Ningxia are arid and semi-arid and are surrounded by the Tengger Desert, the Mu Us Desert, and the Ulan Buh Desert. Ningxia has a desertified land area of 328.67 × 10^4^ ha, which accounts for 65% of the total land area in Ningxia [22], and is a region of China prone to relatively frequent SD storm weather. Yanchi County is an arid region of Ningxia with low precipitation, high winds, copious sand, and severely desertified grasslands, and is a centre within Ningxia that experiences frequent SD storms. The overall climate of Yanchi County showed a trend of warming and drying between 1993 and 2011. However, the grassland vegetation of this region has been rehabilitated for a long time, and the degree of desertification has gradually decreased, owing to the implementation of ecological engineering projects such as the Three-North Shelter Forest Program and the Sloping Land Conversion Program, grazing prohibition within the whole region, and the construction of a sand consolidation demonstration zone [23]. Therefore, the identification of SBAs based on service flow paths and the assessment of the effects of WEP on the SBAs, can demonstrate the spatio-temporal relationship between SPAs and SBAs, which can provide a scientific basis for ecosystem management and the improvement of human life quality and the production environment. This study analysed the spatio-temporal variations of the Yanchi County beneficiary areas of WEP in the following aspects: (1) HYSPLIT was used to simulate flow paths of WEP in terms of dust trajectories and then the trajectories were interpolated to identify beneficiary areas, (2) spatio-temporal correspondences between the SPAs and SBAs were established, and (3) spatio-temporal disparities in the SBAs patterns and the effects of WEP of Yanchi County were analyzed.

## 2. Methodology and Data

### 2.1. Study Area

**Figure 1:**
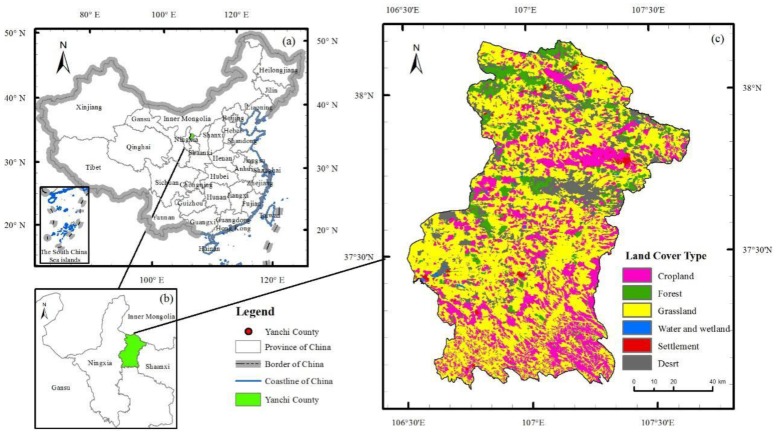
Location and land cover of the study area. (**a**) The position of Yanchi County in China; (**b**) The position of Yanchi County in Ningxia; (**c**) The land cover type of Yanchi County in 2010.

Yanchi County, which is located in the northwest inland (37°04′–38°10′ E, 106°30′–107°41′ N) (Figure 1a) and eastern parts of the Ningxia Hui Autonomous Region (Figure 1b), has a total area of 6777.97 km^2^ and an average altitude of 1600 m. In addition, this county experiences serious desertification and belongs to a typical transition zone. The southern part comprises the Loess Plateau district, while the northern part comprises the hilly region of the Ordos Mesa, with a gentle slope connecting the county with the Mu Us Desert. The climate of Yanchi County is a typical temperate continental climate. The average annual rainfall is 293.1 mm, the annual evaporation is 2403.7 mm, the average wind speed is 2.7 m/s, and the average annual number of wind and sandstorm days are 24.2 days and 20.6 days, respectively. Yanchi County is the centre of the sandstorm region in Ningxia. The total precipitation between March and May is only 51 mm, accounting for 17.4% of the total annual precipitation, which is the main reason for the frequent occurrence of drought and sandstorms in spring. The land cover type of Yanchi County is dominated by grasslands, which accounted for 54.95% of the total area in 2010, followed by croplands and deserts; the county has a sparse distribution of woodlands and water (Figure 1c). The vegetation type is designated as a transition zone type ranging from desert steppe to steppe, and perennial wild herbs are widely distributed across the region. The soil mainly consists of sierozem, eolian sandy soil, black loam, and saline soil. These geographic transitions define the diversity of the region and result in a typical fragile ecological environment.

### 2.2. Methodology

#### 2.2.1. Framework Used to Simulate the Flow of Wind Erosion Prevention

**Figure 2:**
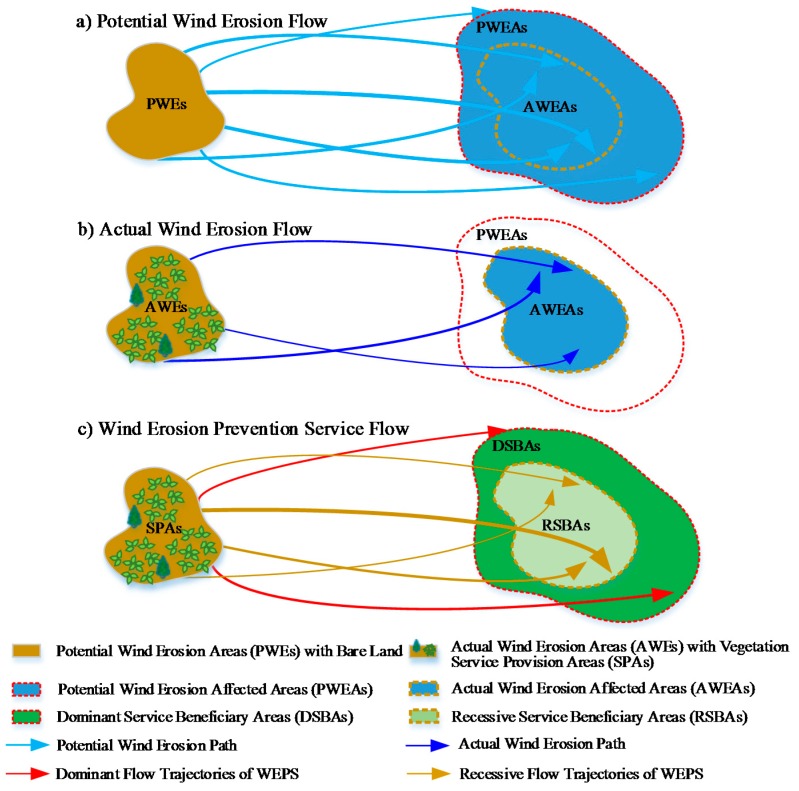
Simplification of service provision areas (SPAs) and service beneficiary areas (SBAs) for wind erosion prevention (WEP) service flow incorporating wind erosion simulation. The simulation of the wind erosion prevention service flow is based on the potential and actual wind erosion service flow. (**a**) In the bare sand land without vegetation, the potential wind erosion flow from the potential wind erosion areas (PWEs) to the potential wind erosion affected areas (PWEAs), which can recognize the maximum wind erosion amount and the maximum wind erosion affected areas. (**b**) In the actual land cover with vegetation, the actual wind erosion flow from the actual wind erosion areas (AWEs) to the actual wind erosion affected areas (AWEAs), which can recognize the actual wind erosion amount and actual wind erosion affected areas. (**c**) The wind erosion prevention service flow can establish the spatial relationship between the SPAs and SBAs. The boundary of the SPAs depends on the areas whose wind erosion prevention amount (the subtraction of potential wind erosion amount and actual wind erosion amount) is positive. The thickness of the light blue and dark blue trajectories represent its carried potential and actual wind erosion amount, respectively. The thicker the path, the more dust it carries. The flow trajectories of the WEP can be divided into two parts, dominant flow trajectories in red, and recessive flow trajectories in brown, which correspond to the dominant service beneficiary areas (DSBAs) and recessive service beneficiary areas (RSBAs), respectively. The naming rule of the dominant and recessive trajectories/areas can be assimilated to the dominant and recessive gene, with the dominant ones corresponding to the dominant gene for their obvious WEP flow effect and the recessive ones corresponding to the recessive gene for their relatively insignificant WEP flow effect. The spatial relationship between the dominant and recessive terms can be better understood from the perspective of ‘set’ in mathematics. The domain of the dominant flow trajectories/DSBAs can be assimilated to the set A and the actual wind erosion service flow trajectories/AWEAs corresponded to complementary set A. The union of the dominant flow trajectories and the recessive flow trajectories is the potential wind erosion paths. The thickness of the red and brown trajectories depend on the subtraction of the potential and actual wind erosion amount of the corresponding trajectory in light blue and dark blue at the same location, representing the wind erosion prevention service flow amount.

The flow of WEP can be traced by the transmission of SD, namely, the movement and settlement process of SD from the source area to the downwind area when the wind speed is greater than or equal to the threshold wind speed that causes SD movement, which is influenced by SD movement conditions and the meteorological conditions that affect the transmission of SD. The threshold wind speed that causes the SD movement differs significantly among different underlying surfaces [24]. The surface roughness for vegetation cover is higher than that for bare land, and the threshold wind speed increases correspondingly. Therefore, the spatial flow simulation of WEP can reflect the influence of the vegetation factors on the flow paths and the range of the beneficiary areas, by distinguishing the difference of threshold wind speeds for different underlying surfaces. Under the bare land condition, potential wind erosion occurs, and the downwind flow path of the potential wind erosion is known as the potential wind erosion path. Actual wind erosion occurs under vegetation cover, and the downwind flow path of the actual wind erosion is the actual wind erosion path (Figure 2). The potential wind erosion path is not less than the actual wind erosion path and contains the actual wind erosion path. The direct benefit of the WEP flow is reflected in the vegetation cover condition, as follows: (1) erosion caused by wind blowing at a speed higher than the threshold wind speed causing SD movement under bare land, but lower than that causing SD movement under vegetation land is prevented; and (2) the amount of SD transported by the actual wind erosion path is lower than that transported by the potential wind erosion path. In this case, the trajectories of the air masses determine the paths of the WEP flow and the beneficiary areas, which can be simulated with HYSPLIT. Because the existing vegetation type is mainly sandy grassland in Yanchi County, the threshold wind speed for sandy grassland is used to calculate the actual wind erosion amount. According to the observation results of the wind and sand activities in Yanchi County [23], the threshold wind speeds of the bare land and sandy grassland are 4.88 m/s and 5.17 m/s, respectively, at a height of 2 m.

#### 2.2.2. Simulation of the Wind Prevention Erosion Flow Paths

In this study, a forward-trajectory analysis was used to determine the flow paths of the WEP [15]. The forward trajectories, which started at an elevation of 500 m above ground level and were centred at the 37.8° N 107.38° E pixel (i.e., Yanchi), were simulated with HYSPLIT every 6 h from 0:00 on 1 January to 24:00 on 31 December in 2010, and the trajectories were only simulated when the maximum 10-min average wind speed in 1 h was greater than or equal to the threshold wind speed. The HYSPLIT model calculated the air mass trajectory using the Lagrangian method [25]. The particles in the air were hypothesized to blow in the wind; therefore, the air mass trajectory was the integral of its position vector in time and space. The final position was calculated by the average velocity of the initial position and the first predicted position.

The first prediction position can be calculated as follows:

(1) $$p^{\prime }\left(t+\Delta t\right)=p\left(t\right)+V\left(p,t\right)\times \Delta t$$

The final position can be calculated as follows:

(2) $$p\left(t+\Delta t\right)=p\left(t\right)+0.5\times \left[V\left(p,t\right)+V\left(p^{\prime },t+\Delta t\right)\right]\times \Delta t$$

where p(t) is the initial position, V(p,t) is the velocity vector of the initial position, Δt is the integral time step, and $V\left(p^{\prime },t+\Delta t\right)$ is the velocity vector of the first guess position.

The integral time step is variable, but the product of the time step and maximum transmission speed should be less than the data grid.

V_max_ × Δ*t* < 0.75 (3)

The meteorological data keeps its original format in the horizontal coordinates, while it is interpolated into the terrain following coordinate system in the vertical direction, as follows:

(4) $$\sigma =\frac{Z_{top}-Z_{msl}}{Z_{top}-Z_{gl}}$$

where *Z_top_* is the top of the trajectory mode coordinate system, *Z_gl_* is the altitude, and *Z_msl_* is the height of the boundary.

#### 2.2.3. Identifying the Beneficiary Areas of the Wind Erosion Prevention Service

Regarding the wind erosion paths, the regions through which the potential wind erosion paths pass are the potential wind erosion affected areas (PWEAs), and the regions through which the actual wind erosion paths pass are the actual wind erosion affected areas (AWEAs). The AWEAs range is completely within the PWEAs scope (Figure 2) and the boundary of the SBAs is same as the PWEAs. The effect of the WEP flow on the beneficiary areas is reflected in the vegetation cover conditions, as follows: (1) for the dominant service beneficiary areas (DSBAs), wind erosion in the part of the PWEAs beyond the AWEAs disappears, and the effect of WEP flow is obvious. (2) For the recessive service beneficiary area (RSBAs), the amount of SD transported in the AWEAs decreases but does not disappear, and the effect of WEP flow is relatively insignificant. The PWEAs, which can be divided into DSBAs and RSBAs, represent the whole beneficiary areas of WEPs (Figure 2).

There are many widely used trajectory statistical methods, such as the residence time analysis (RTA) [26], potential source contribution function analysis (PSCF) [27], quantitative transport bias analysis (QTBA) [28], concentration weighted field (CWT), and residence time weighted concentration (RTWC) [29], which have promoted the study of the pollutant transport path. However, no single method can provide as much information as that obtained by using all of the methods. Li et al. [30] used the statistics of the trajectories to investigate their spatial patterns. In this study, the beneficiary areas were identified using the simulated trajectories of the WEP [31,32]. The trajectories were interpolated onto a 1° × 1° grid with HYSPLIT to obtain the benefit extent, and the value assigned to each grid cell was the frequency with which the trajectories passed through the cell, and was calculated as follows:

(5) $$p_{i}=\frac{L_{i}}{L}\times 100\%$$

where, *p_i_* is the frequency at which the trajectories passed through grid cell *i*, *L_i_* is the number of trajectories that passed through grid cell *i*, and *L* is the total number of trajectories from the starting point. The benefits that people receive from the WEP are reflected by the frequency at which the trajectories pass through a grid cell. For a given grid cell, the higher the frequency, the more benefits people in the grid cell received from the WEP in Yanchi County.

### 2.3. Data

#### 2.3.1. Data Processing

**Figure 3:**
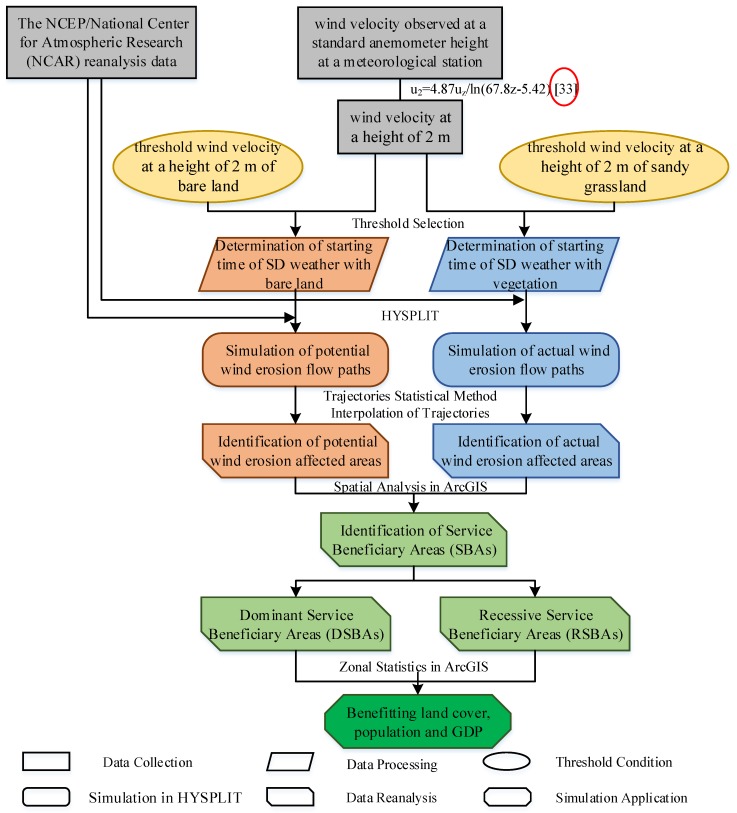
The flow chart of the data processing and related data mining techniques. Note: the shape and color of the box in the flow chart represents the different procedures of data mining. The grey rectangular blocks are the data collection process. The yellow ellipses are the threshold conditions under bare land and vegetation land cover. The rhomboids in orange and blue represent the data processing procedure of the wind speed selection under bare land and vegetation land cover, respectively. The orange and green rounded rectangles are the simulation of the potential and actual wind erosion flow paths under bare land and vegetation land cover, respectively. The light green rectangles without diagonal corners represent the identification of service beneficiary areas based on the spatial analysis in ArcGIS. The dark green rectangle without corners is the process data application. The texts outside the boxes among the arrow’s interval are the related data mining techniques during the data mining process. HYSPLIT—Hybrid Single Particle Lagrangian Integrated Trajectory. (u_2_ = 4.87u_z_ ln(6.87z − 5.42) [33]).

The simulation of the SD flow trajectories in Yanchi County was based on the data processing of meteorological data. This process can be divided into data collection, processing, mining based on the HYSPLIT simulation, statistics, reanalysis, visualization, and application (Figure 3). Firstly, the wind speed records are converted to the wind velocity at a height of 2 m. Secondly, the records that are equal to or exceed the threshold wind speed under bare land and vegetation land cover, respectively, are selected, and the starting time of the SD weather for the HYSPLIT simulation is determined. Thirdly, the flow paths of the wind erosion are simulated in HYSPLIT to extract the meteorological data patterns. Then, the wind erosion affected areas are identified based on the interpolation and statistics of the simulated trajectories. Fourthly, the spatial analysis in ArcGIS (Esri, Redlands, CA, USA) and the spatial relationship of the different service beneficiary areas, defined in Figure 2, are used to identify the SBAs, RSBAs, and DSBAs. Finally, the spatial distribution of benefitting the land cover, population, and gross domestic product (GDP) are determined according to the zonal statistics in ArcGIS.

#### 2.3.2. Data Applied for Simulation and Analysis

To calculate the forward trajectories of dust in Yanchi County, the 10-min maximum wind velocity in 1 h by 6-hourly wind speed records of one day of the Yanchi meteorological station and the National Centers for Environmental Prediction (NCEP)/National Center for Atmospheric Research (NCAR) (which were available from 1948 to the present) reanalysis data in 2010 were needed (Table 1). The 10-min maximum wind velocity in 1 h by 6-hourly wind speed records of one day between 0:00 on 1 January and 24:00 on 31 December in 2010, was from Huayun lnformation Technology Engineering Co., Ltd. (Beijing, China, http://www.huaxin-hitec.com/). The land cover data (spatial resolution of Yanchi County was 30 m and spatial resolution of beneficiary areas was 1 km), population, and GDP data (spatial resolution 1 km) for 2010 were derived from the Data Center for Resources and Environmental Sciences, Chinese Academy of Sciences (CAS) (http://www.resdc.cn).

## 3. Results

### 3.1. Transmission Paths of the Wind Erosion Prevention Service

**Figure 4:**
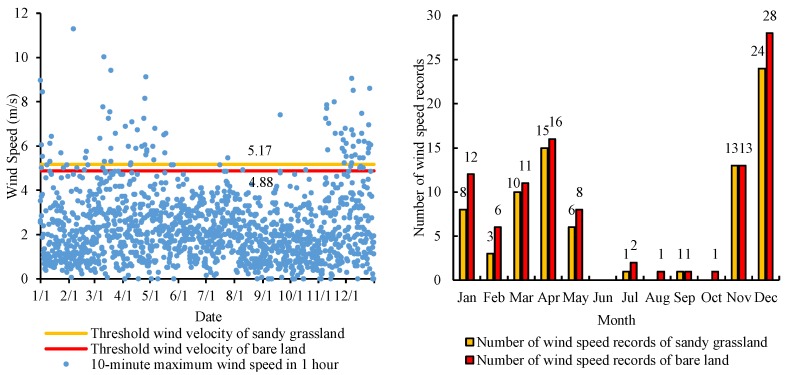
Wind speed and number of wind speed records in each month that equalled or exceeded the threshold wind speed in Yanchi County in 2010.

In 2010, there were 1460 6-hourly wind speed observation records (Figure 4). The maximum wind speed at a height of 2 m in Yanchi County was 11.3 m/s, the minimum wind speed 0 m/s, and the average wind speed 2.3 m/s. There were 99 wind speed records that were greater than the threshold wind speed for the bare land (≥4.88 m/s) under potential wind erosion conditions in 2010. These records mainly appeared in December, April, November, January, and March, and were therefore concentrated in spring and winter. There were 81 wind speed records that were greater than the threshold wind speed for the sandy grassland (≥5.17 m/s) under the actual wind erosion conditions in 2010. These records mainly appeared in December, April, November, and January, and were therefore also concentrated in spring and winter. On the whole, the wind speeds in December and April were relatively high, creating the basic conditions for wind erosion.

**Figure 5:**
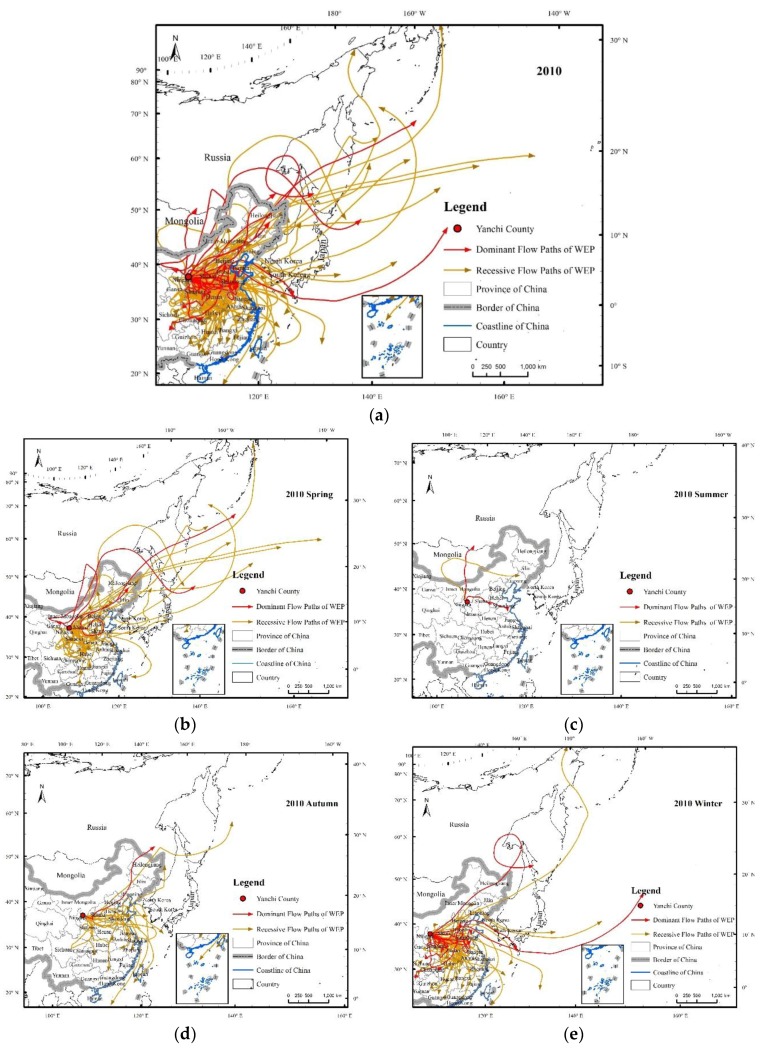
Flow paths of the WEP service by the ecosystems in Yanchi County in 2010. The flow paths represent the trajectories of dust simulated by Hybrid Single Particle Lagrangian Integrated Trajectory (HYSPLIT) when the wind speeds were ≥5.17 m/s for the sandy grassland and ≥4.88 m/s for the bare land, respectively, and they extended over a large area (**a**). We classified the flow paths into two categories according to their wind erosion influence to the downwind areas, dominant flow trajectories of the WEP (the red arrow) and recessive flow trajectories of the WEP (the bronze arrow). All of the paths can be separated according to their occurring season, spring (from March to May) (**b**), summer (from June to August) (**c**), autumn (from September to November) (**d**), and winter (from December to February) (**e**).

The simulated SD transmission paths in 2010 mainly passed through central and eastern China, North Korea, South Korea, Japan, Mongolia, and eastern Russia (Figure 5), indicating that the SBAs of WEP in Yanchi County extended far beyond China. In China, the SD transmission paths of Yanchi County mainly flowed through Shaanxi, Shanxi, Hebei, Shandong, Beijing, Tianjin, Henan, Hubei, Jiangsu, and Liaoning. Li et al. [34] indicated that a sandstorm in Ningxia not only caused serious damages and great losses in the local area, but also affected the environments in Japan, the United States, and other regions. The downwind transmission paths in Ningxia can be divided into a northwestern path (Ningxia–Inner Mongolia–Hebei–Beijing–North Korea–Japan–United States) and a northern path (Ningxia–Shaanxi–Shandong–Pacific), which are consistent with the results of this study. The SD transmission paths represented the flow of the WEP from Yanchi County (SPAs) to its downwind areas (SBAs). In 2010, the dominant flow paths of the WEP amounted to 18, among which 2 occurred in spring (March to May), 2 in summer (June to August), 1 in autumn (September to November), and 11 in winter (December to February), which were located in the northeastern, northern and eastern, northeastern, and northeastern to southwestern Yanchi County, respectively. There were 81 recessive WEP flow paths, and of these, 31 occurred in spring (March to May), 1 occurred in summer (June to August), 14 occurred in autumn (September to November), and 35 occurred in winter (December to February); the flow direction of these flow paths was basically located approximately between the northeast and southeast of Yanchi County. Overall, the transmission paths of the WEP were more intensive and the transmission distance was farther in spring and winter than in summer and fall in 2010. Simultaneously, with the increase in the transmission distance, the intensity of the path decreased, indicating that the WEP flow had seasonal and spatial proximity characteristics. This type of temporal distribution is mainly influenced by vegetation and meteorological conditions [35]. Under the influence of the east Asian monsoon, 75% of the precipitation occurs in summer and autumn, and the wind erosion in Yanchi County usually occurs in spring and winter. In addition, the vegetation cover in summer and autumn is usually higher than that in spring and winter.

### 3.2. Beneficiary Areas of the Wind Erosion Prevention Service

Before the identification of the SBAs of WEP, it is necessary to distinguish between the PWEAs and the AWEAs. In 2010, the areas of the PWEAs and AWEAs were 1153.2 × 10^4^ km^2^ and 968.1 × 10^4^ km^2^, respectively; of these, the PWEAs and AWEAs within China had areas of 392.4 × 10^4^ km^2^ and 357.1 × 10^4^ km^2^, covering 40.9% and 37.2% of the total area of China, respectively (Table 2). These results showed that the wind erosion affected the area of Yanchi County beyond the Chinese border and the proportion of the influence area in China were relatively large. The influence of wind erosion in Yanchi County had transboundary and transregional characteristics. The wind erosion in spring had the largest influence area, and the SD weather was more frequent in spring than in other seasons.

**Figure 6:**
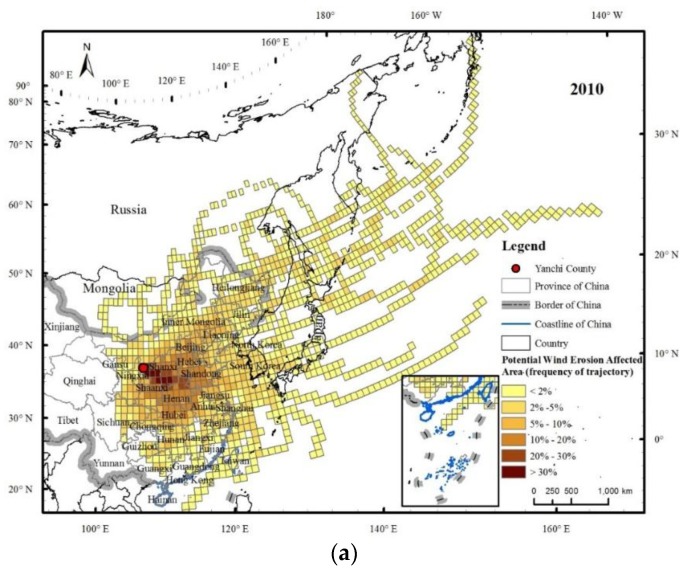

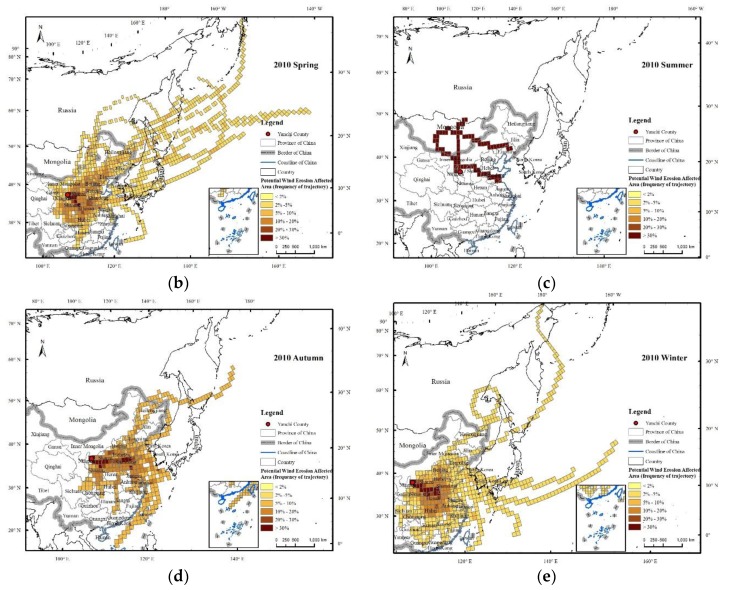
Potential wind erosion affected areas of Yanchi County in 2010 (**a**) and their patterns in different seasons, including spring (**b**), summer (**c**), autumn (**d**) and winter (**e**). Note: the value of each grid cell is the ratio of the number of potential wind erosion simulated trajectories passing through the grid cell to the total number of simulated trajectories. These values represent the potential wind erosion amount that accrued to the people in each grid cell; higher values (shown in darker colours) imply that the people living in those grid cells derived greater wind erosion risks.

**Figure 7:**
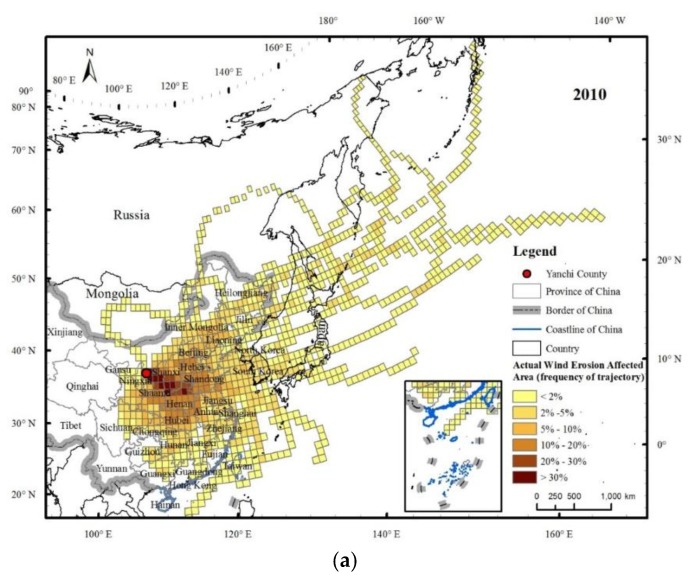

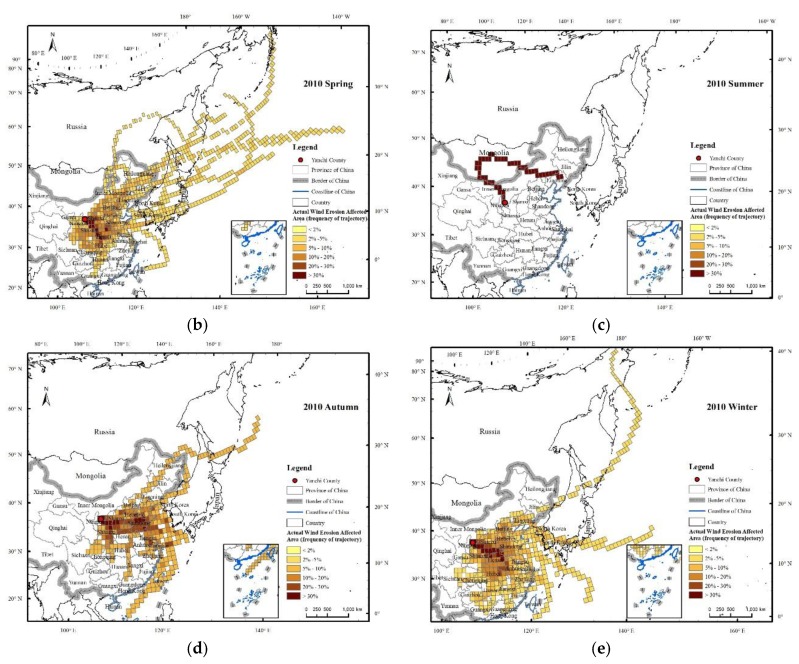
Actual wind erosion affected areas of Yanchi County in 2010 (**a**) and their patterns in different seasons, including spring (**b**), summer (**c**), autumn (**d**) and winter (**e**). Note: the value of each grid cell is the ratio of the number of actual wind erosion simulated trajectories passing through the grid cell to the total number of simulated trajectories. These values represent the actual wind erosion amount that accrued to the people in each grid cell; higher values (shown in darker colours) imply that the people living in those grid cells derived greater wind erosion risks.

The PWEAs and AWEAs had similar spatial distribution patterns (Figure 6 and Figure 7), and were mainly located in eastern and central China, North Korea, South Korea, Japan, eastern Russia, and eastern Mongolia. The areas through which the flows passed with a high frequency (≥10%) were mainly in Shaanxi, Shanxi, Henan, western Shandong, Hebei, Beijing, and northern Hubei, among which the trajectory frequency of the Yan’an district, north-central Shaanxi, and Linfen in the southwestern Shanxi Province, exceeded 30%. The influence area spatial scale varied with obvious seasonal changes, and the influence in spring was the most widespread, extending to areas of the central and eastern parts of China and to North Korea, South Korea, Japan, eastern Russia, and even Alaska in the United States. This spatial scale was the second largest in winter, with affected areas as far as North Korea, South Korea, Japan, and eastern Russia. Regarding the transmission direction, the spatial pattern of the wind erosion affected areas in spring was similar to that in the whole year. The wind erosion affected areas were concentrated in the southeastern Yanchi County, while the areas in summer were much smaller and mainly located in the eastern region of Yanchi.

**Figure 8:**
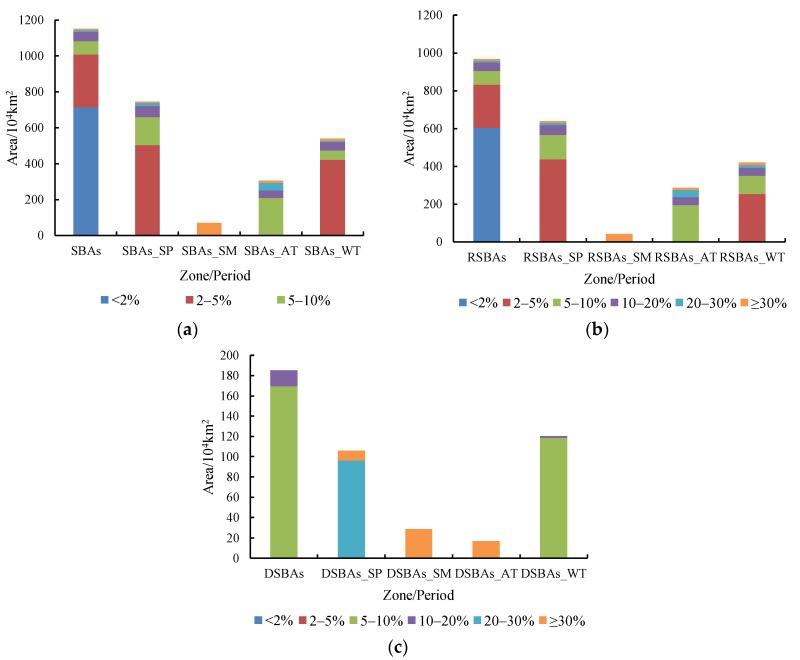
Statistical areas of different path distribution frequencies in SBAs (**a**), RSBAs (**b**), and DSBAs (**c**). Note: the frequency categories are same as the standards in Figure 6 and Figure 7. The abscissa labelled with SP, SM, AT, and WT represent the corresponding beneficiary areas in spring, summer, autumn, and winter, respectively.

**Figure 9:**
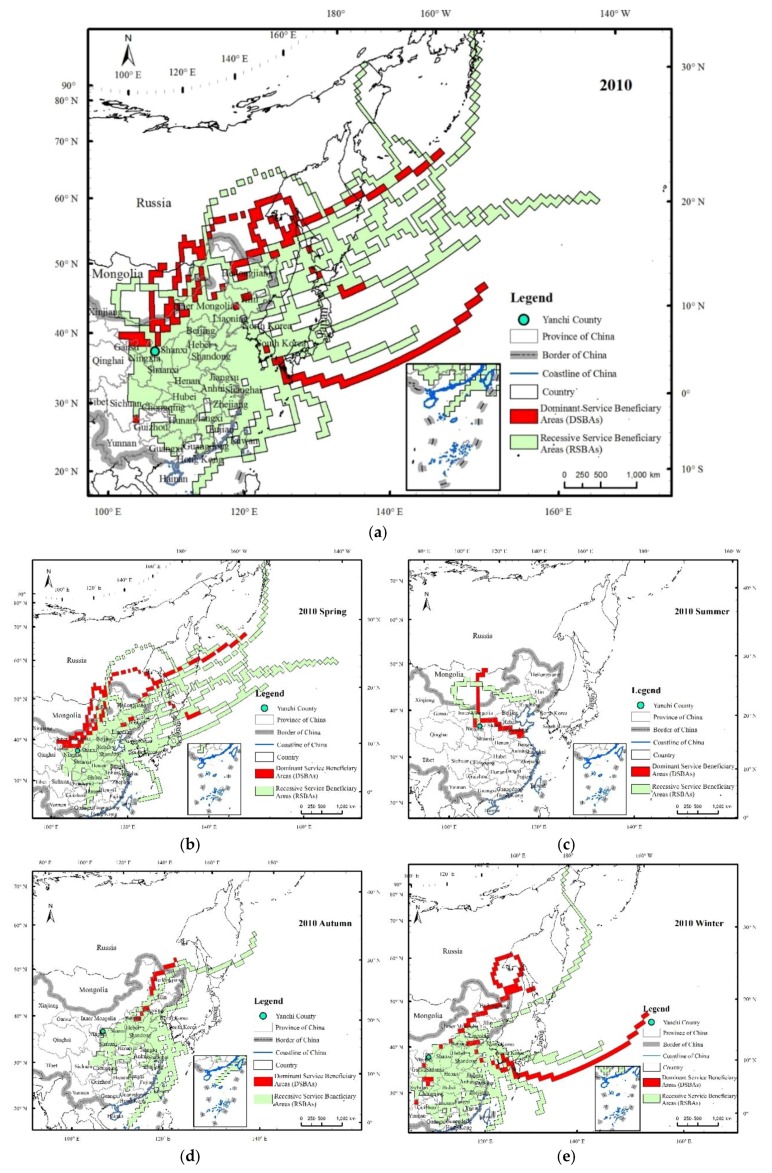
The DSBAs and RSBAs of Yanchi County’s WEP service in 2010 (**a**), and their patterns in different seasons, including spring (**b**), summer (**c**), autumn (**d**) and winter (**e**).

According to the influence of the WEP on the downwind beneficiary areas, the PWEAs are defined as all of the areas benefiting from the WEPS, namely, the SBAs, which can be divided into DSBAs and RSBAs, and the difference between the DSBAs and RSBAs is due to the WEP flow of Yanchi County. There is no actual wind erosion in the DSBAs, and, although actual wind erosion can still occur in the RSBAs, the wind erosion amount decreases, which is a direct reflection of the effect of the WEP flow. The area of the DSBAs in 2010 was 185.1 × 10^4^ km^2^ in 2010 (Table 2), accounting for 16.1% of the total DSBAs area; of these, the DSBAs within China comprised 35.4 × 10^4^ km^2^, accounting for 19.1% of the total DSBA areas and 3.7% of the total area of China. According to the distribution frequency of the WEP flow paths, 91.5% of the DSBAs were in the region with the distribution frequency of 5% to 10% (Figure 8). The distribution patterns of the trajectory frequencies varied both spatially and seasonally. Spatially, the trajectory frequency distribution pattern of the RSBAs was similar to that of the SBAs, with most of the areas in the region with a distribution frequency less than 2%. From the standpoint of temporal change, the trajectory frequency distribution patterns in spring and winter were similar, and most areas of the RSBAs and SBAs in spring and winter were in the region with a frequency distribution of 2% to 5%. The DSBAs were mainly distributed in the marginal zone of the RSBAs (Figure 9), as the transmission paths in the central SBAs were more densely distributed, and the dominant and recessive transmission paths overlapped in space by interpolation. In 2010, the DSBAs were mainly distributed in the north of the beneficiary areas, namely, the border between Inner Mongolia and eastern Mongolia, eastern Russia, Kyushu, and the northeastern sea of Kyushu. The DSBAs in spring were the largest, and the spatial distribution of these DSBAs was similar to that of the DSBAs for the whole year. Furthermore, the DSBAs in spring were mainly located in northeastern China, eastern Russia, southeastern South Korea, Kyushu, and the northeast coast of Japan and had trajectory frequencies mainly between 20% and 30%. Combined with the frequency of the wind erosion transmission paths in the beneficiary areas, the frequency of the paths decreased gradually with the increase in the distance from Yanchi County, and the effect of the WEP flow decreased correspondingly. As a whole, the WEP flow had the greatest effect in the east and southeast regions of Yanchi County.

### 3.3. Land Cover Type in Areas Benefitting from the Wind Erosion Prevention Service

In terms of the service flow paths distribution frequencies, the distribution frequencies of the overseas regions were mostly below 5%, and the effect of the WEP flow was not significant. Therefore, this study only analysed the flow effects of the WEP in the SBAs within China. In 2010, the SBAs of Yanchi County’s WEP in China covered an area of 392.4 × 10^4^ km^2^ (Table 2). In descending order, these SBAs accounted for cropland (35.09%); forest (29.67%); grassland (21.52%); desert (6.72%); settlement (4.18%); and wetland and water (2.81%), equivalent to 77.19% of China’s cropland, 51.95% of China’s forest, 28.22% of China’s grassland, 13.25% of China’s desert, 83.14% of China’s settlement, and 39.94% of China’s wetland and water regions. According to the distribution frequency of the WEP flow paths, the proportion of the grassland with a distribution frequency of greater than 30% in 2010 was the largest, and the proportion of cropland with a distribution frequency of 2% to 30% was the largest (Table 3).

**Figure 10:**
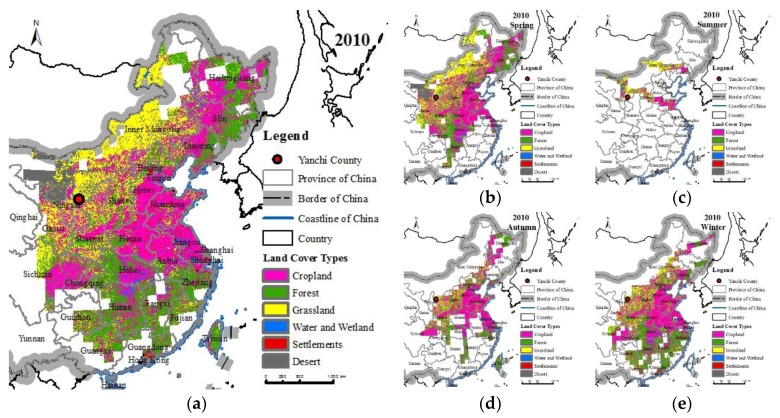
Benefitting land cover in China from Yanchi County’s WEP service in 2010 (**a**), and their patterns in different seasons, including spring (**b**), summer (**c**), autumn (**d**) and winter (**e**).

The benefitting cropland was mainly in the North China Plain, Northeast Plain, Jianghan Plain, and Dongting Lake Plain, which contained almost all of the major grain producing areas in China (Figure 10). The benefitting forest was mainly in the Changbai Mountain region in the northeast, southeastern Hunan, Zhejiang, Fujian, Guizhou, Guangxi, and Jiangxi. The benefitting grassland was in Inner Mongolia. The benefitting water and wetlands were located in the middle and lower reaches of the Yellow River and in the middle and lower reaches of the Yangtze River. The main benefitting settlements were located in Shaanxi, Shanxi, Henan, western Shandong, Hebei, Beijing, northern Hubei, and in the regions of rural settlements scattered around cropland. There were also seasonal differences in the benefitting land cover types with changes in the SBAs (Table 4, Figure 10). The range of benefitting cropland and forest areas was the largest in winter and second largest in spring, and this cropland was mainly distributed in the North China Plain. The effect of the WEP flow was insignificant in winter in North China, because winter is not a growing season, while the opposite was observed in spring. The benefitting forest in winter was mainly located in southeastern China, including Zhejiang, Fujian, Guangdong, Hunan, and Guizhou, and the effects of the WEP flow reached a certain degree of influence in both spring and winter. The grassland had the largest beneficiary areas in spring, which were mainly located in Inner Mongolia, and the WEP flow effect was consequently highest in spring. For the wetland and water body regions, the benefit of the WEP flow in winter in the north was relatively low. For the settlements and deserts, there was no obvious seasonal difference in the benefits of the WEP flow. The distribution of the DSBAs in spring was similar to that in the whole year, that is, the DSBAs were mainly located in the border area of Inner Mongolia and eastern Mongolia, and were mainly grassland. In contrast, the main land cover type was cropland, in summer, autumn, and winter.

## 4. Discussion

### 4.1. Human Welfare Associated with the Wind Erosion Prevention Service

**Figure 11:**
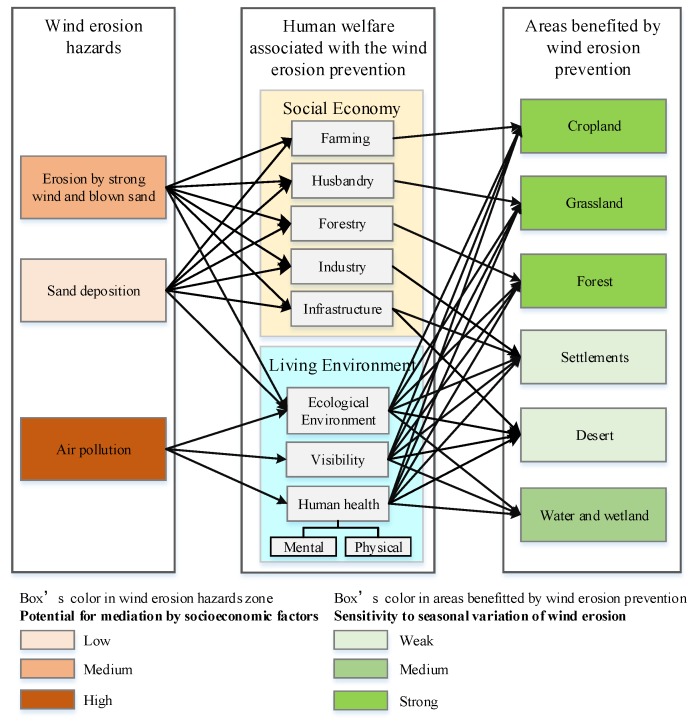
Framework used to identify the beneficiary areas of the WEP service.

The hazards produced by sandstorms along the wind erosion trajectories include erosion by strong wind and sand flow, sand deposition, and air pollution. Wind erosion prevention by vegetation reduces the hazards associated with sandstorms and produces benefits for the people living along the wind erosion trajectories. Social economic benefits include reduced damage to farming, animal husbandry, and forestry production, and the benefits to living environments include reduced damage to infrastructure (e.g., traffic, water conservancy facilities, construction, and wireless systems) and human health, as well as the prevention of reduced visibility; the areas where these benefits are realized include forest, grassland, cropland, settlements, and water and wetland regions (Figure 11). The effect of sandstorms on health has become a major concern in recent years. Chan et al. [36] found that acute exposure to Asian dust transported long range can increase the number of non-accidental and cardiovascular deaths for people of all ages on dust storm days in Taipei, Taiwan. In addition, the particulate matter with aerodynamic diameters less than 10 and 2.5 μm (PM_10_ and PM_2.5_) showed a statistically significantly increase of 24.2 μg/m^3^ and 7.9 μg/m^3^ per dust day, respectively, on the dust days compared to the reference days. Furthermore, epidemiological studies have established a robust association between acute and chronic exposure to PM_2.5_ and adverse health effects, and have suggested that Asian dust storms increase emergency room visits for ischaemic heart diseases, cerebrovascular diseases and chronic obstructive pulmonary diseases [37], and daily hospital admissions for intracerebral haemorrhagic strokes [38], and have caused specific mortality, cardiovascular and pulmonary diseases, asthma, and lung cancer [39,40,41]. Therefore, the WEP service, which can prevent wind erosion and the movement of SD to downwind areas, is beneficial to public health.

**Figure 12:**
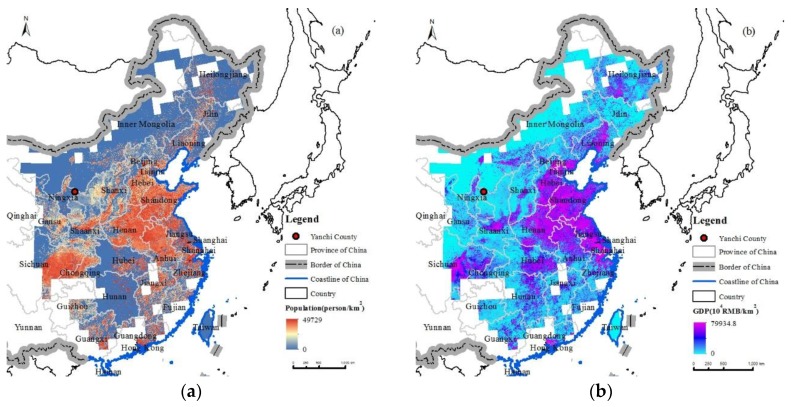
Benefitting population (**a**) and GDP (**b**) in China from Yanchi County’s WEP service in 2010.

The benefitting population was identified from the population distribution data and the grid cells through, which the simulated trajectories passed, and the GDP of these cells increased because of the reduced damage to farming, animal husbandry, and forestry production, due to the prevention of wind erosion. In 2010, the total population in the SBAs was approximately one billion, accounting for 77.11% of the total population of China (Table 5). The number of benefitting people in the DSBAs was estimated to be 1.58% of the total population of China, with an average population density of 58 people·km^−2^, and the number of benefitting people in the RSBAs was estimated to be 75.36% of the total population of China, with an average population density of 276 people·km^−2^. The benefitting population suffered less public health risks from the wind erosion in Yanchi County. The total GDP of the SBAs was 26.8 trillion RMB (RMB is an abbreviation of Chinese currency name, i.e., Ren Min Bi, and as of 2018-06-22, 6.497 RMB = 1 US $), accounting for 87.90% of the total GDP of China in 2010; of these, the total GDP of the DSBAs accounted for 1.73% of the total GDP of China in 2010, with an average GDP density of 1.50 million RMB·km^−2^, while the total GDP of the RSBAs was 85.58% of the total GDP of China in 2010, with an average GDP density 7.32 million RMB·km^−2^. The DSBA populations and socio-economic development were no longer affected by wind erosion, because of the WEP flow of Yanchi County. The frequency at which the trajectories passed through a grid cell were treated as a proxy for the benefit of the WEP experienced by the people in the cell. The higher the grid cell values, the more benefits the people received from the prevention of wind erosion by the Yanchi County ecosystems. From the perspective of the WEP flow path distribution frequency, the population density and GDP density were the highest in the region, with a frequency of 10–20%, with values of 468 person·km^−2^ and 11.2 million RMB·km^−2^, respectively; this region was mainly located in Hebei, Shandong, Henan, and Beijing (Figure 12). The above region comprised provinces and cities with a high economic development level and large population density. The total population and GDP in the region, with a frequency of 2–5%, reached 337 million people and 10.07 trillion RMB, respectively, accounting for 25.82% of China’s total population and 33.04% of China’s GDP in 2010.

### 4.2. Implications for Ecosystem Service Value Flow and Eco-Compensation

To improve the ecological environment and attain a sustainable development, grassland grazing prohibitions were first implemented in Yanchi County in 2002, including a number of large-scale trials and demonstrations of grass grid sand-fixation, the return of farmland to grassland, vegetation restoration in degraded grassland, and the breeding and replanting of improved sandy degraded grassland. These measures benefitted the improvement of the service function and the ecological environment in Yanchi County. However, there were inevitable conflicts between the ecological policies and the economic interests of farmers and herdsmen [11], and 70% of the farmers and herdsmen were illegally grazing without permission [42]. The opportunity costs of development incurred by farmers and herdsmen to protect the ecosystem should be borne by the downwind SBAs, and the ecological compensation should compensate for the shortage of ecological protection funds in Yanchi County. This study identified the transmission paths and SBAs of the WEP, based on the wind erosion flow paths, and established the spatio-temporal relationships between SPAs (the ecosystems in Yanchi County) and SBAs. However, there was no quantification of the diffusion of the WEP amounts. The spatial diffusion process of the WEP was also the diffusion process of the WEP value. Therefore, the WEP value flow process can be identified based on the existing service flow process, combined with ecosystem service values, which can provide a direct scientific basis for the formulation of the ecological compensation standards and have important theoretical and practical significances.

### 4.3. Uncertainty Analysis

This study established a spatio-temporal relationship between the SPAs and the SBAs of the WEP in Yanchi County, and clarified the spatio-temporal disparities of the SBAs. However, there were some uncertainties and constraints in this study. The WEP services represent a kind of protective service, and the WEP function always takes effect under actual vegetation cover conditions. In addition, the quality of the atmospheric environment is the result of multiple factors, including vegetation, soil properties, and air pollution. Therefore, the benefits of the WEP can only be assessed by simulation. The identified beneficiary areas are the potential maximum affected areas, and we cannot monitor the effect of the protective service. The simulation assumed that there was a sufficient enough sand source to be blown when the wind speed was greater than or equal to the threshold wind speed, to simplify the flow path of SD without considering the instability of the atmospheric junction. The transmission of the SD can only occur when the following three conditions are met: sufficient sand source, strong wind, and unstable atmosphere. In the future, these three conditions should be included in the simulation of the flow path of the WEP. Because of the data limitations, the wind speed data of a meteorological station in Yanchi was used to simulate the SD movement of the whole Yanchi County, and the simulated trajectories represented the flow paths of the WEP without considering the spatial heterogeneity of the meteorological data. Future research should consider the appropriate simulation scale for different quantities of meteorological data, and explore the mechanisms and effects of the WEP flow more deeply, in order to build a better relationship between the WEP flow and public health.

## 5. Conclusions

The ES flows emphasize the recognition of real SBAs from the perspective of human welfare. This study analysed the SD transport path by using the HYSPLIT model, based on meteorological data, including the 10-min maximum wind velocity and the NCEP/ NCAR reanalysis data in 2010. Then, the study identified the spatio-temporal disparities of the SBAs based on the WEP flow, and further divided the SBAs into DSBAs and RSBAs to clarify the benefitting land cover, benefitting people, and GDP distribution. The results showed the following: (1) There were 99 flow paths (including 18 dominant flow paths and 81 recessive flow paths) of the WEP in 2010, mainly extending to central and eastern China, North Korea, South Korea, Japan, Mongolia, and eastern Russia. Temporally, the transmission paths were more intensive and longer in spring and winter than in summer and autumn, and the intensity of the paths decreased as the transmission distance increased, indicating that the flow of the WEP had seasonal and spatial proximity characteristics. (2) In 2010, the area of the SBAs of the WEP in Yanchi County was 1153.2 × 10^4^ km^2^, for which the area of the DSBAs was 185.1 × 10^4^ km^2^ and was mainly distributed in the marginal zone of the RSBAs. The areas through which the flow paths passed with a high frequency (≥10%) were mainly located in Shaanxi, Shanxi, Henan, western Shandong, Hebei, Beijing, and northern Hubei. The spatial scale of these areas varied with obvious seasonal changes, and the seasonal influence in spring was the greatest. As a whole, the flow effect of the WEP in the east and southeast regions of Yanchi County was the largest. (3) The SBAs of the WEP within China covered an area of 392.4 × 10^4^ km^2^ in 2010, with the largest proportion comprising cropland. According to the distribution frequency of the flow path of the WEP, the proportion of grassland in the region with a distribution frequency exceeding 30% in 2010 was the largest, and the proportion of cropland was the largest in the region with a distribution frequency of 2% to 30%. (4) In 2010, the total number of people benefitting from the WEP of Yanchi County reached one billion, accounting for 77.11% of the total population of China, and the total GDP of the SBAs was 26.8 trillion RMB, accounting for 87.90% of the total GDP of China. The population and social and economic development in the DSBAs (1.58% of the total population of China, 1.73% of the total GDP of China) were no longer affected by wind erosion. This study established the spatio-temporal relationships between the SPAs and SBAs, and revealed spatio-temporal disparities among the areas benefitting from the WEP. This analysis framework can be applied to other research areas and has an important theoretical significance to the study of ESs flow, which can provide a scientific basis for ecological protection policy formulation and ecological compensation standard accounting.

## Figures and Tables

**Table 1:** The wind velocity records that equal or exceed the threshold wind speed of the bare land (4.88 m/s) at the height of 2 m in Yanchi County in 2010 (unit: m/s).

Time (Hour–Day–Month)	10-min Maximum Wind Velocity in 1 h	Time (Hour–Day–Month)	10-min Maximum Wind Velocity in 1 h	Time (Hour–Day–Month)	10-min Maximum Wind Velocity in 1 h	Time (Hour–Day–Month)	10-min Maximum Wind Velocity in 1 h	Time (Hour–Day–Month)	10-min Maximum Wind Velocity in 1 h
6–1–1	8.98	6–10–3	7.78	6–25–4	8.15	12–10–11	7.85	6–9–12	5.98
6–2–1	6.06	6–11–3	10.02	0–26–4	9.13	6–11–11	5.83	18–9–12	6.51
18–2–1	5.01	6–12–3	5.31	6–26–4	5.61	6–12–11	7.03	6–11–12	5.61
0–3–1	5.53	6–16–3	7.26	6–27–4	5.76	6–18–11	8.00	18–15–12	6.21
6–3–1	8.45	6–18–3	7.55	12–27–4	5.83	6–19–11	6.58	6–16–12	5.46
6–8–1	5.16	0–19–3	5.31	6–4–5	5.98	6–26–11	6.58	18–16–12	5.01
6–11–1	6.13	6–19–3	5.91	0–5–5	5.39	6–27–11	5.91	6–17–12	5.09
18–11–1	5.31	12–19–3	9.42	6–6–5	6.81	6–28–11	6.28	6–18–12	7.48
6–13–1	6.43	6–24–3	6.58	6–16–5	6.51	6–29–11	5.24	18–18–12	6.58
18–23–1	5.68	6–4–4	6.88	6–17–5	5.68	18–29–11	5.76	6–20–12	5.46
6–26–1	5.01	6–7–4	5.98	6–18–5	6.58	6–30–11	6.81	18–20–12	5.01
6–30–1	5.16	18–7–4	5.98	12–24–5	5.16	0–1–12	5.53	6–21–12	6.21
6–6–2	11.29	12–9–4	5.16	0–26–5	5.16	6–1–12	6.13	6–25–12	5.68
18–10–2	5.01	18–10–4	5.24	12–16–7	5.16	12–1–12	5.53	0–26–12	6.13
6–18–2	4.94	12–11–4	7.11	12–25–7	5.46	18–6–12	5.09	6–26–12	6.96
6–19–2	5.46	6–15–4	5.91	12–10–8	4.94	0–7–12	6.73	18–26–12	5.91
6–20–2	5.76	6–17–4	6.73	12–20–9	7.40	6–7–12	9.05	6–27–12	8.60
6–22–2	5.16	6–22–4	5.98	6–18–10	4.94	12–7–12	5.53	12–28–12	6.06
12–7–3	6.36	18–24–4	7.26	6–9–11	7.26	18–7–12	5.24	18–28–12	6.06
0–10–3	5.01	0–25–4	6.06	6–10–11	7.70	0–9–12	8.53		

Note: the format of the time is hour–day–month. For example, 6–2–1 represents the time of 6 o’clock on 2 January. There were 99 wind speed records that were greater than the threshold wind speed for bare land (≥4.88 m/s) under the potential wind erosion condition, and 81 wind speed records that were greater than the threshold wind speed for sandy grassland (≥5.17 m/s) under the actual wind erosion condition in 2010.

**Table 2:** The seasonal variation in the wind erosion affected areas and service beneficiary areas (SBAs) of wind erosion prevention (WEP) of Yanchi County in 2010 (unit: 10^4^ km^2^). DSBAs—dominant service beneficiary areas; RSBAs—recessive service beneficiary areas; AWEAs—actual wind erosion affected area; PWEAs—potential wind erosion affected areas.

Period	Within China			Overseas		
	DSBAs	RSBAs/AWEAs	PWEAs/SBAs	DSBAs	RSBAs/AWEAs	PWEAs/SBAs
2010	35.35	357.07	392.42	149.77	611.01	760.78
Spring_2010_	32.98	253.83	286.80	73.10	385.27	458.36
Summer_2010_	20.24	20.36	40.60	8.69	21.42	30.11
Autumn_2010_	13.05	145.14	158.19	3.95	142.54	146.49
Winter_2010_	27.19	238.78	265.97	93.07	182.66	275.73

**Table 3:** Land cover benefitting from Yanchi County’s WEP service in 2010, analysed by trajectory frequency (unit: 10^4^ km^2^).

Trajectory Frequency	Cropland	Forest	Grassland	Water and Wetland	Settlements	Desert
<2%	22.28	44.40	24.46	1.94	2.17	11.94
2–5%	48.31	39.87	31.77	5.10	4.89	11.37
5–10%	29.71	16.59	14.88	2.30	2.30	2.16
10–20%	29.62	12.51	7.91	1.47	4.65	0.16
20–30%	5.60	1.94	2.15	0.17	0.83	0.32
≥30%	2.47	1.36	3.45	0.07	0.09	0.48

**Table 4:** Land cover benefitting from Yanchi County’s WEP service in 2010, analysed by season (unit: 10^4^ km^2^).

Period	Cropland	Forest	Grassland	Water and Wetland	Settlements	Desert
2010	138.00	116.69	84.63	11.06	16.45	26.43
spring_2010_	104.08	66.42	74.10	7.69	12.42	22.65
summer_2010_	11.30	3.32	15.31	0.77	1.68	8.22
autumn_2010_	72.71	42.12	24.08	5.69	10.35	4.07
winter_2010_	109.50	87.63	42.23	8.18	14.03	5.26

**Table 5:** Population and gross domestic product (GDP) benefitting from Yanchi County’s WEP service in 2010.

Frequency of Trajectory	Benefitting Area (10^6^ km^2^)	Ratio to the Total Area of China (%)	Benefitting Population Density (People·km^−2^)	Benefitting Population (Million People)	Ratio to the Total Population of China (%)	Benefitting GDP Density (Million RMB·km^−2^)	Benefitting GDP (Trillion RMB)	Ratio to the Total GDP of China (%)
<2%	1.07	11.15	145	154.98	11.89	3.83	4.10	13.44
2–5%	1.41	14.69	239	336.69	25.82	7.14	10.07	33.04
5–10%	0.69	7.22	277	191.98	14.72	7.09	4.92	16.13
10–20%	0.56	5.87	468	263.82	20.23	11.21	6.31	20.71
20–30%	0.11	1.15	451	49.57	3.80	10.86	1.19	3.92
≥30%	0.08	0.82	106	8.37	0.64	2.56	0.20	0.66
In sum	3.9	40.90	281	1005.41	77.71	7.12	26.80	87.90
